# Supplementary material for: A Comparative Study of the Synthesis and Characterization of Biogenic Selenium Nanoparticles by Two Contrasting Endophytic Selenobacteria
Source: Microorganisms. 2023 Jun 16;11(6):1600. doi: 10.3390/microorganisms11061600 (PMC10303461; doi:10.3390/microorganisms11061600)
Supplement: Supplementary file 1 [file microorganisms-11-01600-s001.zip › microorganisms-2399419-supplementary.pdf]

microorganisms

Supplementary materials

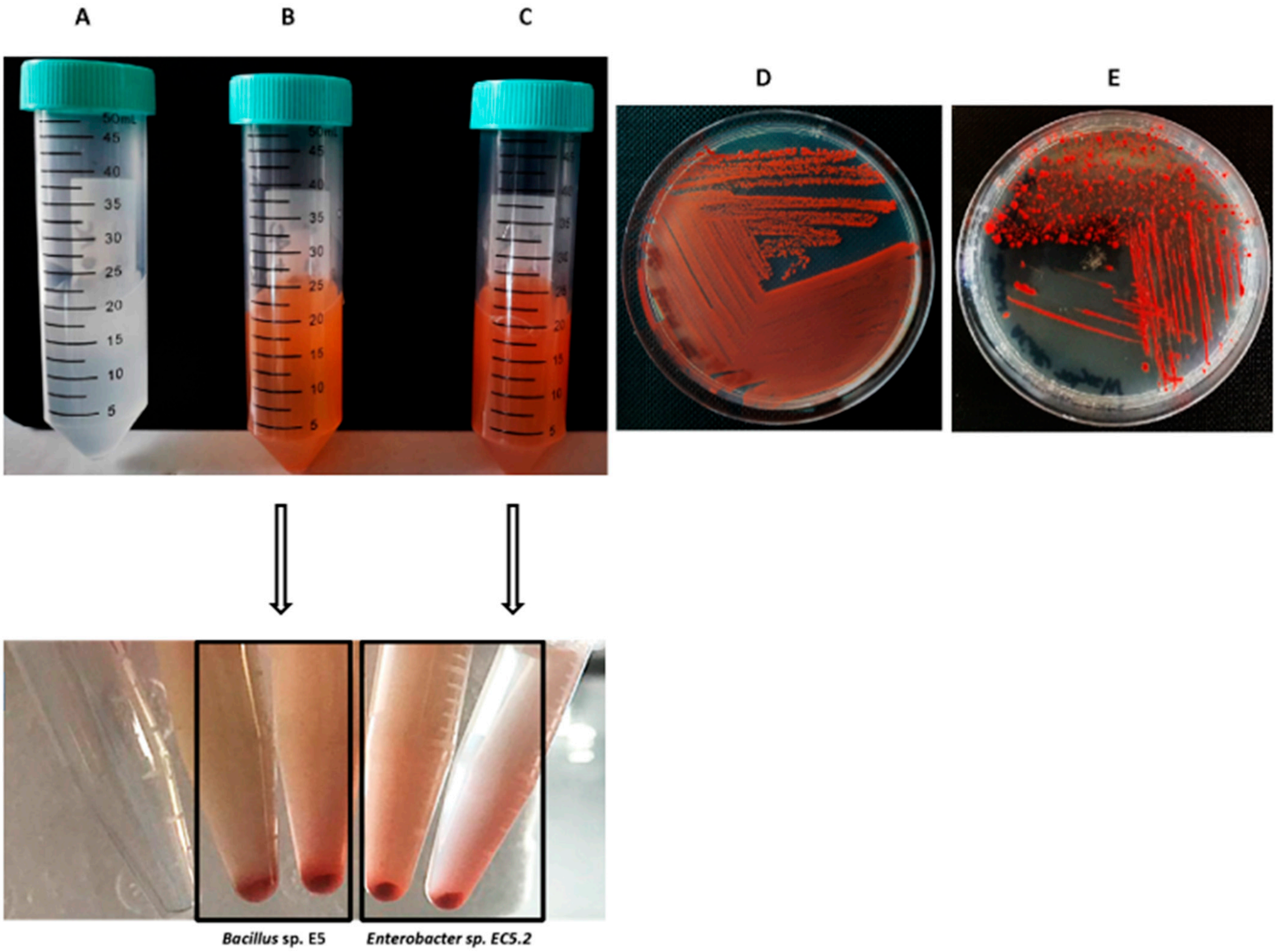

Figure S1. Growth of *Bacillus* sp. E5 and *Enterobacter* sp. EC5.2 in Tris-HCl pH 8 in the presence of selenite (A= without selenite; B= *Bacillus* sp. E5; C= *Enterobacter* sp. EC5.2). Liquid (A, B, C) and solid medium (D= *Enterobacter* sp. EC5.2; E= *Bacillus* sp. E5) turned to red only in the presence of 5 mM selenite. Images were obtained after culturing for 6 h (A, B, C) and 24 h (D, E).

A

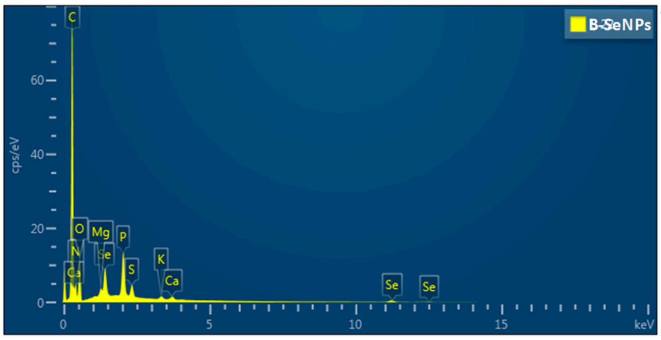

Element	Weight %	Weight % Sigma
N	28.14	0.97
O	46.68	0.74
Mg	1.41	0.09
P	9.66	0.19
S	2.91	0.09
K	0.76	0.07
Ca	0.93	0.07
**Se**	**9.51**	**0.24**
Total	100.00	

B

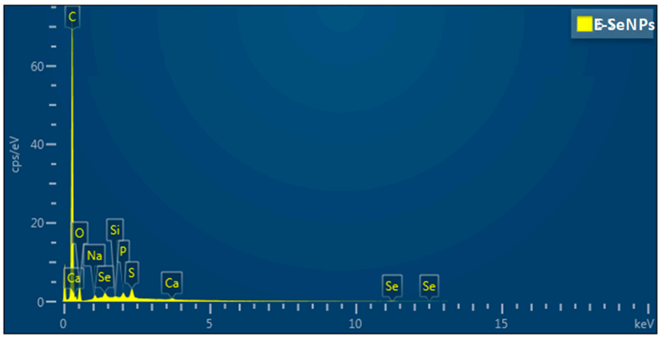

Element	Weight %	Weight % Sigma
O	60.53	0.77
Na	6.55	0.41
Si	1.09	0.26
P	6.80	0.33
S	13.31	0.38
Ca	2.14	0.27
**Se**	**9.58**	**0.60**
Total:	100.00	

Figure S2. EDS graph of selenium nanoparticles (SeNPs) biosynthesized by *Bacillus* sp. E5 (A) and *Enterobacter* sp. EC5.2 (B) with 5mM of Na_2SeO_3 for 6h.

Table S1. Total Se content in bacterial biomass of two endophytic bacterial strains (*Bacillus* sp. E5 and *Enterobacter* sp. EC5.2) isolated from wheat plants, grown with and without Se supplementation.

Bacterial strain	Se (mM)	Total Se (mg kg^{-1})
***Bacillus* sp. E5**	0	40 ± 14
Enterobacter sp. EC5.2	0	9 ± 3
***Bacillus* sp. E5**	5	12016 ± 4205
***Enterobacter* sp. EC5.2**	5	3543 ± 1240

Table S2. FTIR absorption bands and identification of *Bacillus* sp. E5 and *Enterobacter* sp. EC5.2 unchallenged cells, and biogenic samples (B-SeNPs, E-SeNPs).

$\tilde{\nu}$ (cm^{-1})	Vibrational modes	Identification
3280	ν (O–H; N–H)	Amide A (Proteins) [79]
2927	ν_{as} (C–H in > CH2)	Lipids [79]
1625	ν (N-H)	Amide I (proteins) [80]
1529	ν (C-N)	Amide II (proteins) [79]
1450	δ (–CH3)	Proteins, lipids, polyesters, etc. [73]
1390	ν_s (COO–)	Amino acid side chains; lipids; peptides [75]
1233	δ (C–OH)	carboxylic acids [75]
1054	ν (CC); ν (CO)	Polysaccharides [75]

Where ν and δ indicate stretching and bending, respectively; *as* and *s* stand for scissoring and out of phase in plane vibrations.
